# Comparison of Survival and Safety Between Total Omentectomy and Partial Omentectomy for Gastric Cancer: A Meta-Analysis

**DOI:** 10.3389/fsurg.2021.708545

**Published:** 2021-12-24

**Authors:** Yue-Xin Zhang, Han-Dong Liu, Ze-Hua Chen, Tao Jin, Jian-Kun Hu, Kun Yang

**Affiliations:** ^1^Department of Gastrointestinal Surgery, West China Hospital, Sichuan University, Chengdu, China; ^2^Laboratory of Gastric Cancer, State Key Laboratory of Biotherapy/Collaborative Innovation Center of Biotherapy and Cancer Center, West China Hospital, Sichuan University, Chengdu, China; ^3^Department of Gastrointestinal Surgery, West China Hospital Sichuan University Jintang Hosptial, Chengdu, China

**Keywords:** total omentectomy, partial omentectomy, gastric cancer, survival, safety

## Abstract

**Background:** The greater omentum can limit abdominal inflammation and act as a protective cushion, but it is always involved in dissemination of gastric cancer. The purpose of this meta-analysis was to compare the survival and safety between total omentectomy and partial omentectomy for gastric cancer.

**Methods:** Two investigators independently conducted a systematic search of PubMed, Embase, CNKI, and Cochrane Library ranging from January 2000 to November 2020. The pooled odds ratio (ORs) and weighted mean difference (WMD) with the 95% confidence interval (95% CI) were used to assess perioperative and survival parameters.

**Results:** A total of 2,031 patients in 11 studies (574 patients in the partial omentectomy group and 1,457 patients in the total omentectomy group) were included. The results found shorter operation time (WMD = −25.584; *P* = 0.000) and less intraoperative blood loss (WMD = −47.301; *P* = 0.050) in the partial omentectomy group, compared to total omentectomy. There were no significant differences in terms of incidence of complications (OR = 0.770; *P* = 0.164), blood transfusions rates (OR = 0.269; *P* = 0.161), time to first flatus (WMD = 0.160; *P* = 0.345), hospital stay (WMD = −1.258; *P* = 0.087), and number of harvested lymph nodes (WMD = 1.265; *P* = 0.662). For the disease-free survival (OR = 0.80; *P* = 0.381) and overall survival, there were no statistical differences between the two procedures.

**Conclusions:** The partial omentectomy could reduce operation time and trended to decrease intraoperative blood loss. And the survival in patients with partial omentectomy seemed to be comparable to that of patients with total omentectomy.

## Introduction

Gastric cancer is one of the most common cancers in the world. Surgery takes the core position of treatment of gastric cancer; the prognosis of patients with gastric cancer is closely correlated to the radicality of resection of gastric cancer.

Complete resection of the greater omentum was considered to be the key to ensuring the elimination of micrometastasis (1), because the greater omentum is closely associated with the intraperitoneal spread of gastrointestinal malignant tumors (2, 3). And lymph node metastasis in the greater omentum could lead to recurrence (4), whereas partial omentectomy (PO) might compromise the lymph node dissection. As a mesenteric tissue, however, the greater omentum contains a large number of immune cells, which helps to prevent intestinal adhesion and eliminate abdominal inflammation (5). Other advantages of preserving the greater omentum include shortening operation time and reducing complications, especially in the popular laparoscopic gastrectomy where the risks of spleen and colon injury were greatly increased (6). On the other hand, a prospective, observational cohort study [OMEGA trial (7)] included 100 patients with gastric cancer with total omentectomy (TO), and it found rare patients had positive omental metastasis in the postoperative pathological report. And if there was micrometastasis of gastric cancer cells in the greater omentum, the clinical stage of the patients should be stage IV, and total omentectomy cannot improve the survival of patients theoretically. Therefore, whether patients can actually benefit from total omentectomy, especially for advanced cases and patients receiving laparoscopic gastrectomy, remains unclear. Meanwhile, clinical guidelines across the world related to omentectomy during gastrectomy are inconsistent. The latest NCCN gastric cancer guideline recommended the removal of both the greater omentum and the lesser omentum (8), but the European guidelines did not give any recommendations for omentectomy (9). According to current Japanese guidelines for gastric cancer, partial omentectomy can be performed in T1 and T2 tumors, but this was not the standard procedure for T3 or deeper tumors (10).

Therefore, the purpose of this meta-analysis was to evaluate the differences in survival, complications, operation time, and operation related parameters between total omentectomy and partial omentectomy in gastric cancer patients, aiming at guiding the surgical treatment of gastric cancer and pointing out the direction of future research.

## Methods

This meta-analysis was conducted by the reporting guidance to PRISMA (11). Since identifiable patient personal information was not involved, this systematic review and meta-analysis did not require ethical approval.

### Search Strategy

Two investigators individually searched the PubMed, Embase, CNKI, and Cochrane databases using the terms (stomach OR gastric) AND (cancer OR carcinoma) AND (omentectomy OR omentum-preserving OR omentum-preserved OR omentum resection), regardless of language. The electronic search was up to November 2020. The controlled trials comparing total omentectomy to partial omentectomy in gastric cancer surgery were eligible. Conference data and gray literatures were also included if any.

### Inclusion Criteria

The inclusion criteria for the studies were as follows:

1. Patients with gastric adenocarcinoma;
2. Study should include data comparing total omentectomy and partial omentectomy in patients with gastric cancer;
3. The included patients should receive radical surgery and appropriate lymph node dissection;
4. Either distal gastrectomy or total gastrectomy was included;
5. Either early cancer or advanced cancer was included;
6. Either open surgery or laparoscopic surgery was included;
7. Essential information such as postoperative comorbidities, mortalities or survival outcomes were reported.

### Exclusion Criteria

Studies that met the following criteria were excluded:

1. Studies without control groups;
2. Case reports, reviews, letters, and comments;
3. Lack of necessary data for statistical analysis;
4. The pathological types of cancer cells in the patients were gastric stump cancer, lymphoma, and neuroendocrine tumor etc.;
5. The patients in the literature received neoadjuvant chemotherapy;
6. Patients with distant metastasis, such as peritoneal seeding and liver metastasis.

### Data Extraction and Quality Assessment of the Studies

Two researchers independently extracted data from each study, including publication year, author's name, country, stage of tumor, sample size, the type of operation, operation time, mortality, morbidity, and postoperative survival outcomes. The Newcastle-Ottawa Scale (NOS) was used to assess the methodological quality of non-RCT studies. The NOS evaluated the study based on the selection of study groups, comparability between groups, and exposure/outcomes. Studies with a score of ≥6 were considered to be high quality (12). The Cochrane risk of bias tool was used to assess the methodological quality of RCT studies (13).

### Data Analysis

We used the STATA version 12.0 (StataCorp., College Station, TX) to analyze. The heterogeneity between the studies was tested by Cochran's Q and Higgins's *I*^2^ statistics. If there was no heterogeneity (*I*^2^ < 50%, *P* > 0.10), a fixed-effect model was used. If the heterogeneity existed, we adopted three methods to explain heterogeneity: (1) using random-effect model; (2) subgroup analysis stratified by the operation method (laparoscopic vs. open surgery) or stages (early gastric cancer vs. advanced gastric cancer); (3) sensitivity analysis by excluding studies one by one. Dichotomous variables were measured using the odds ratio (OR), and continuous variables were evaluated to obtain the weighted mean difference (WMD) with 95% confidence intervals (CIs). *P* < 0.05 was defined as statistically significant. If the published paper only provides the median, range, and size of the sample, we estimated the mean and variance by transforming the formula (14).

## Results

**Figure 1:**
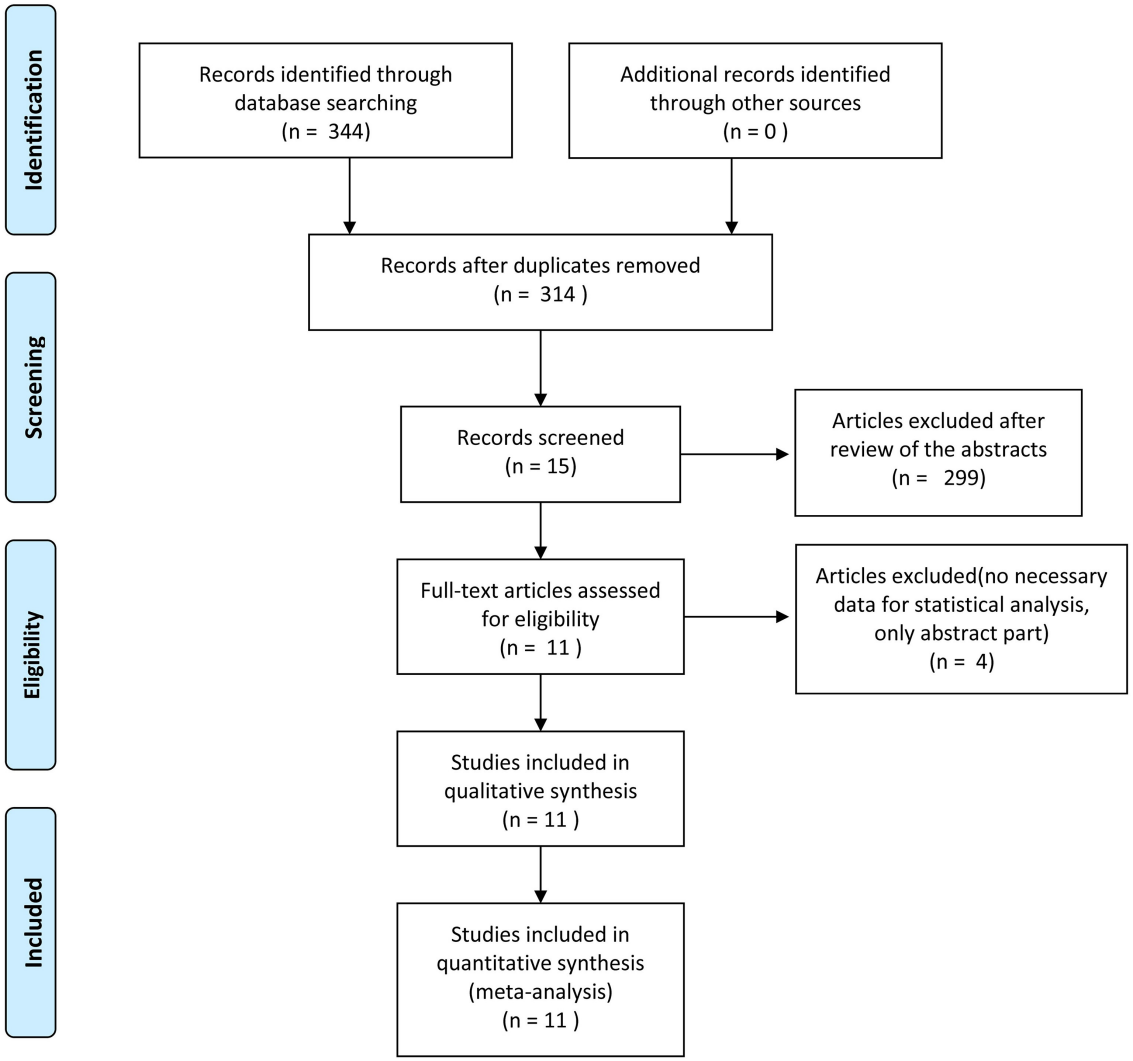
Flow chart for study selection.

The flow chart of search strategy is shown in Figure 1. A total of 344 articles were identified after searching the database. After deleting duplicate records, there were 314 articles left. A total of 299 articles were excluded after reviewing the titles and abstracts. Four articles were furtherly excluded since lack of essential data. Finally, 11 articles were included in the analysis. The publication date ranged from 2011 to 2020. According to the NOS, five articles received a score of 5, one article was scored 6, and four articles were scored 8. Ten studies were retrospective cohort studies (2, 15–23). One RCT (24), which was evaluated by the Cochrane risk of bias tool, was a conference abstract from ASCO. The RCT had a high risk of bias, mainly due to lack of detailed information, and the biases are expected to be reduced after full publication. The characteristics and quality assessment of included studies are presented in Table 1. A total of 2031 patients were included. With respect to the stage of retrieved cases, eight articles which included 1,443 cases focused on early gastric cancer, and the rest discussed advanced gastric cancer. A total of 574 patients and 1,457 patients underwent partial omentectomy and total omentectomy respectively (Table 1).

**Table 1:** Characteristics of patients between total omentectomy and partial omentectomy group.

**Author**	**Year**	**Country**	**Study design**	**Stage**	**Group**	**Cases**	**Age range (yr)**	**Gender (male/female)**	**Surgical procedure**	**Follow-up**	**Quality Scores**
Wang et al. (17)	2012	China	NRCT	EGC	PO TO	20 20	58.6 ± 10.1 58.2 ± 9.7	32 / 8	Open	NA	5/9
Chen Tao et al. (19)	2014	China	NRCT	EGC	PO TO	26 26	62.34 ± 0.13 63.52 ± 1.17	33 / 19	NA	NA	5/9
Wan et al. (21)	2016	China	NRCT	EGC	PO TO	33 33	NA	37 / 29	NA	NA	6/9
Chen Deman et al. (22)	2014	China	NRCT	EGC	PO TO	25 25	54.2 ± 6.5 56.2 ± 7.8	34 / 16	NA	NA	5/9
Zhai et al. (18)	2015	China	NRCT	EGC	PO TO	36 36	55.56 ± 1.16 55.62 ± 1.23	43 / 29	NA	NA	5/9
Ye et al. (20)	2014	China	NRCT	EGC	PO TO	5 5	NA	5 / 5	Open	NA	5/9
Kim et al. (15)	2011	Korea	NRCT	EGC	PO TO	17 20	58.6 ± 10.1 58.2 ± 9.5	28 / 9	Open	NA	8/9
Ha et al. (16)	2008	Korea	NRCT	EGC	PO TO	124 992	56.3 ± 11.3 57 ± 11.3	768 / 348	NA	NA	8/9
Kim et al. (23)	2014	Korea	NRCT	AGC	PO TO	66 80	62.2 ± 11.0 60.9 ± 11.2	106 / 40	laparoscopic	5 years	8/9
Hasegawa et al. (2)	2013	Korea	NRCT	AGC	PO TO	98 98	NA	144 / 52	Open / laparoscopic	5 years	8/9
Yamada et al. (24)	2020	Japan	RCT	AGC	PO/TO	256	NA	NA	Open	NA	High Risk

### Outcome Measures

Detailed information is shown in Table 2.

**Table 2:** Outcome of survival, safety, operation-related events and subgroup analysis stratified by the operation method or stages.

	**No. of studies**	PO (n^*^/N)	TO (n^*^/N)	**OR/WMD (95% CI)**	**P-value for effect size**	**P-value for heterogeneity**	**Effect model**
5-year relapse-free survival	2	125/164	130/178	0.80 (0.48,1.32)	0.381	0.284	Fixed
Complications	9	74/402	89/418	0.77 (0.53,1.11)	0.164	0.833	Fixed
The number of retrieved lymph nodes	4	228^†^	242^†^	1.27 (−4.40,6.93)	0.662	0.023	Random
Intraoperative blood loss (ml)	5	287^†^	284^†^	−47.3 (−94.78,0.08)	0.050	0.000	Random
Operation time (min)	9	504^†^	1372^†^	−25.6 (−37.3, −13.9)	0.000	0.000	Random
Time to first flatus (d)	4	68^†^	71^†^	0.16 (−0.173,0.49)	0.345	0.994	Fixed
Postoperative hospital-stay (d)	6	256^†^	1127^†^	−1.26 (−2.7,0.184)	0.087	0.000	Random
Blood infusion	3	0/62	4/65	0.269 (0.043,1.69)	0.161	0.949	Fixed
**Stratified by stages**							
Complications^#^	6	5/114	12/118	0.43 (0.15,1.21)	0.108	0.723	Fixed
Intraoperative blood loss (ml)^#^	3	64^†^	64^†^	−24.32(−79.0,30.4)	0.384	0.068	Random
Operation time (min)^#^	7	281^†^	1152^†^	−21.9 (−31.6, −12.2)	0.000	0.000	Random
The number of retrieved lymph nodes^#^	2	37^†^	40^†^	6.95 (0.88,13.01)	0.025	0.987	Fixed
Complications^+^	3	69/288	77/300	0.85 (0.57,1.26)	0.405	0.707	Fixed
Operation time (min)^+^	2	223^†^	220^†^	−39.8 (−133, −13.8)	0.406	0.000	Random
Intraoperative blood loss (ml)^+^	2	223^†^	220^†^	−109.4 (−94.7,0.08)	0.058	0.000	Random
The number of retrieved lymph nodes^+^	2	191^†^	202^†^	−2.65 (−6.06,0.77)	0.129	0.136	Fixed
**Stratified by the operation method**							
Operation time(min)^&^	3	162^†^	162^†^	−19.75 (−30.5, −8.9)	0.000	0.200	Fixed
Intraoperative blood loss(ml)^&^	2	130^†^	127^†^	−0.29 (−13.38,12.8)	0.966	0.283	Fixed
Time to first flatus(d)^&^	2	22^†^	25^†^	0.182 (−0.38,0.74)	0.524	0.893	Fixed
Complications^&^	2	1/71	3/85	0.437 (0.056,3.39)	0.429	0.512	Fixed

* *Represents the patients alive;*

† *The summed number of patients in each group;*

# *Represents that the gastric cancer stage of the patient is early gastric cancer;*

+ *Represents that the gastric cancer stage of the patient is advanced gastric cancer;*

& *Represents the operation method of open surgery; OR, Odds ratio; WMD, Weighted mean differences; CI, Confidence interval; PO, Partial omentectomy; TO, Total omentectomy; NA, Not applicable*.

#### Survival

The survival rates of patients with gastric cancer, especially those with advanced gastric cancer, was the primary outcome. Only two articles provided postoperative survival information, but we were merely able to conduct a meta-analysis on 5-year relapse-free survival, because of the difference in survival indexes; we found no difference in 5- ear relapse-free survival between TO and PO (OR = 0.799; 95% CI: 0.484, 1.320; P = 0.381). Furthermore, Hasegawa et al. (2) focused on 3-year relapse-free survival and 3- and 5-year overall survival and found no statistical difference between TO and PO for advanced gastric cancer patients.

#### Complications

**Figure 2:**
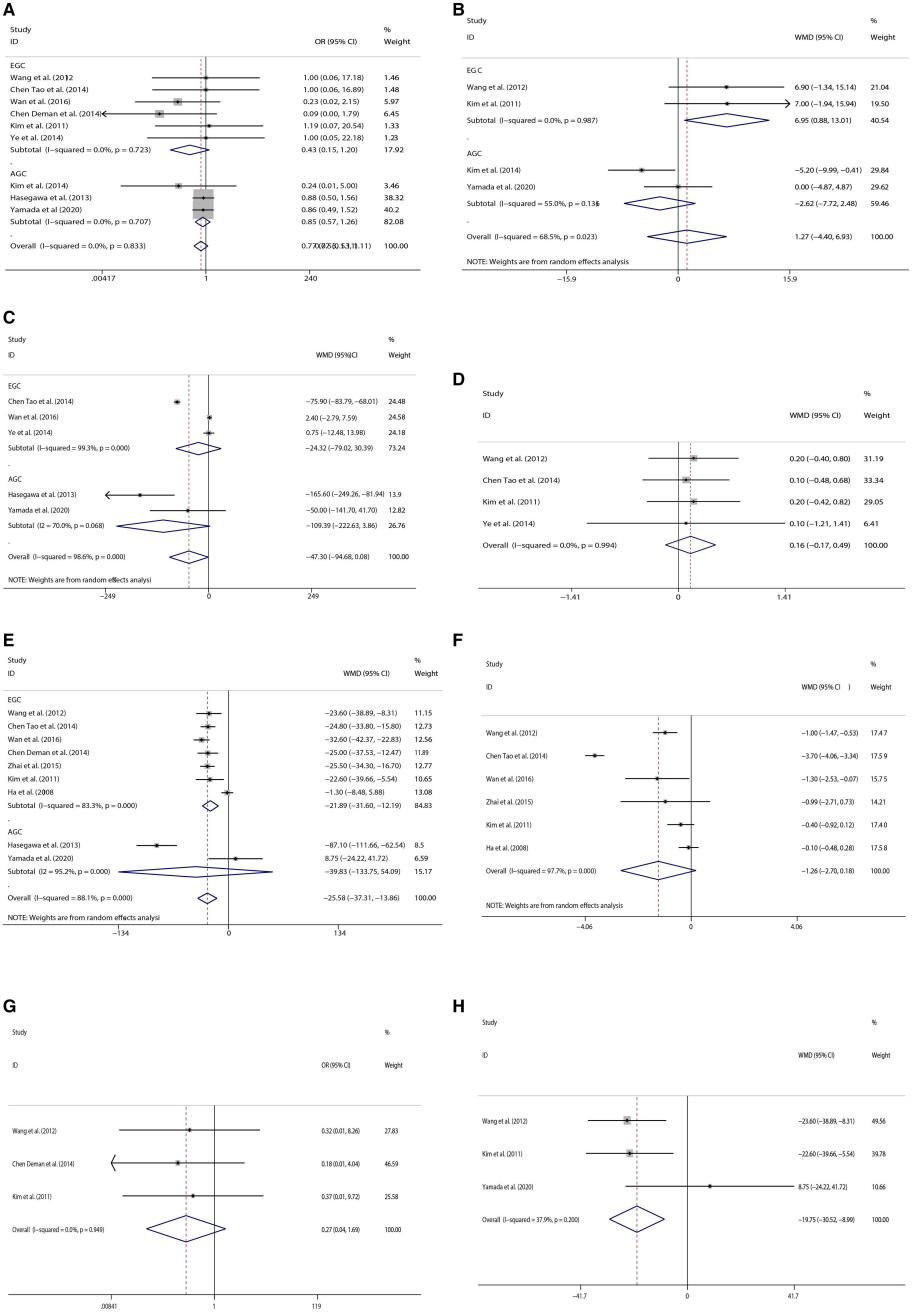
Forest plots[**(A)** Complications, **(B)** The number of retrieved lymph nodes, **(C)** Intraoperative blood loss, **(D)** Time to first flatus, **(E)** Operation time, **(F)** Postoperative hospital stay, **(G)** Blood Infusion, **(H)** Comparison of operation time in open surgery].

Nine studies including six on early cancer and three on advanced cancer provided 163 cases in total to further analysis on complications regardless of detailed types of complications. There was no significant difference in the incidence of complications between the two surgical procedures (OR = 0.770; 95% CI: 0.533, 1.113; *P* = 0.164) (Figure 2A).

#### The Number of Retrieved Lymph Nodes

Four studies provided the number of retrieved lymph nodes. Two types of omentectomy seemed to provide no difference in the number of retrieved lymph nodes (WMD = 1.265; 95% CI: −7.716, 2.476; *P* = 0.662) (Figure 2B).

#### Intraoperative Blood Loss

Five studies provided data on intraoperative blood loss. Although there was no significant difference in intraoperative blood loss between the two kinds of omentectomy, partial omentectomy trended to reduce intraoperative bleeding (WMD= −47.301; 95% CI: −94.683, 3.961; *P* = 0.050) (Figure 2C).

#### Time to First Flatus

Four studies provided the time when the patient began to flatus after operation. Two types of omentectomy seemed to show no difference in the recovery of intestinal movement (WMD = 0.160; 95% CI:−0.173, 0.493; P = 0.345) (Figure 2D).

#### Operation Time

Nine studies were analyzed by random-effects model, for the reason of heterogeneity (*I*^2^ = 88.1%, *P* = 0.000). The operation time of PO was less than TO (WMD = −25.584; 95% CI: −37.308, −13.860; *P* = 0.000) (Figure 2E).

#### Postoperative Hospital-Stay

Six studies focused on early gastric cancer were analyzed by random-effects model. There was no significant difference in length of hospital stay between the two types of omentectomy (WMD = −1.258; 95% CI: −2.701, −0.184; *P* = 0.087) (Figure 2F).

#### Blood Infusion

For patients with early gastric cancer, three studies gave the data on whether patients received blood transfusions during the operation. The result showed similar blood transfusions rates between PO and TO (OR = 0.269; 95% CI: 0.043, 1.687; *P* = 0.161) (Figure 2G).

### Subgroup Analysis

We performed the subgroup analysis stratified by stages (early gastric cancer vs. advanced gastric cancer). There were no significant differences in the incidence of complications (OR = 0.428; 95% CI: 0.152, 1.205; *P* = 0.108) and intraoperative blood loss (WMD = −24.32, 95% CI: −79.02, 30.39, *P* = 0.384) in patients with early gastric cancer, but partial omentectomy cost less time (WMD = −21.89; 95% CI: −31.60, −12.19; *P* = 0.000), and retrieved more lymph nodes (WMD = 6.95; 95% CI: 0.88, 13.01; *P* = 0.025) than total omentectomy. For patients with advanced gastric cancer, there were no significant differences between the two kinds of omentectomy in the incidence of complications (OR = 0.85; 95% CI: 0.57, 1.26; *P* = 0.405), operation time (WMD = −25.58, 95% CI: −133.75, 54.09, *P* = 0.406), and the number of retrieved lymph nodes (WMD = −2.65, 95% CI: −6.06, 0.77, *P* = 0.129). Furthermore, partial omentectomy trended to reduce intraoperative bleeding, although there was no significant difference in intraoperative blood loss between the two kinds of omentectomy (WMD = −109.4, 95% CI: −94.7, 0.08, *P* = 0.058).

Moreover, we also conducted the subgroup analysis stratified by the operation method (laparoscopic vs. open surgery). Only five articles included identified surgical procedures, four of which were performed by open surgery and one by laparoscopy. However, the four studies with open surgery reported different clinical parameters, and we were able to perform the meta-analysis on operative time, intraoperative blood loss, time to first flatus, and the incidence of complications. After analyzing four studies that provided essential information, we found that partial omentectomy cost less time than total omentectomy (WMD = −19.754, 95% CI: −30.517, −8.992, *P* = 0.000, Figure 2H), but we could not find any differences in intraoperative blood loss (WMD = −0.285, 95% CI: −13.383, 12.812, *P* = 0.966), time to first flatus (WMD = 0.182, 95% CI: −0.377, 0.741, *P* = 0.524), and the incidence of complications (OR = 0.437; 95% CI: 0.056, 3.399; *P* = 0.429).

### Sensitivity Analysis

**Figure 3:**
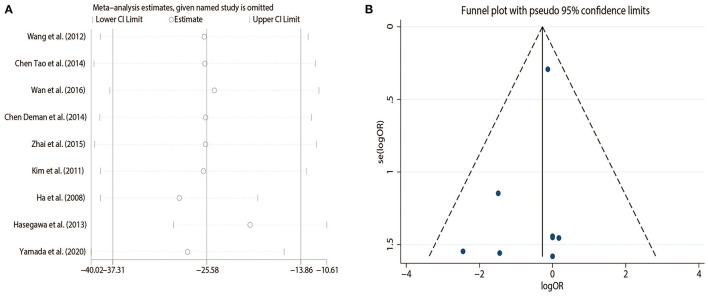
**(A)** Sensitivity analysis on the operation time, **(B)** The funnel plot on the complications.

Sensitivity analysis showed that the conclusion remained unchanged after excluding each study one by one (Figure 3A).

### Publication Bias

The funnel plot on the complications is presented in Figure 3B and shows that there was no evidence of publication bias.

## Discussions

Since the metastatic rate of omental lymph nodes and possibility of omental micrometastasis of cancer cells are low in early gastric cancer, total omentectomy was not considered to provide more benefit than partial omentectomy for patients with T1/T2 gastric cancer. However, limited research focused on the comparison between partial omentectomy and total omentectomy in T3/T4 gastric cancer. Theoretically, total omentectomy seemed to offer the possibility of more complete lymph node dissection and elimination of micrometastasis of greater omentum in advanced gastric cancer. However, our study showed that total omentectomy had no obvious advantages in retrieving the lymph nodes. And the survival analysis showed that 5-year relapse-free survival was not higher for patients receiving TO than for those receiving PO. Since the included studies were limited, additional studies in the future about the impact of partial omentectomy on the extent of lymph node dissection and long-term survival are needed.

Considering the anatomical location and tissue composition of the greater omentum, total omentectomy is prone to injure the colon and spleen, while the omentum promotes angiogenesis through release of fibroblast growth factors and hence has a role in healing inflamed or ischemic tissue and reducing complications such as abdominal abscess, ascites, anastomotic leakage, intestinal obstruction, wound infection, and iatrogenic damage (25–27). However, our study could not draw the conclusion that partial omentectomy has lower complications than total omentectomy for early gastric cancer and advanced gastric cancer patients. The possible reason might be because there were differences in the complexity of the operation process among the patients and both of the two procedures could be safely performed by experienced skillful surgeons. As the result of RCT, there was no significant difference in the incidence of complications between the two procedures in patients with advanced gastric cancer. The safety between the TO and PO needs to be further verified. Nevertheless, partial omentectomy had shorter operation time and tendency of less intraoperative blood loss compared to total omentectomy, especially in the more complicated and time-wasting laparoscopic surgery, although there was only one study on laparoscopic study in the present meta-analysis.

There were some limitations in our study. Firstly, the number of relevant studies, especially large-scale RCT studies, were limited, and the sample sizes for the pooled meta-analyses were relatively small. In addition, the numbers of published studies on advanced gastric cancer or laparoscopic gastrectomy were inadequate. Hence, our findings must be interpreted and generalized cautiously. Secondly, data about the recurrence of gastric cancer and long-term survival outcomes was insufficient. More studies investigating the long-term prognosis of gastric cancer patients between TO and PO are needed. Thirdly, some data was obtained through indirect methods, such as some means and standard deviations from the median and interquartile range, which can impair the accuracy of results. Although limitations were inevitable, we made efforts to minimize the biases by developing a detailed protocol, performing a cautious search, using objective methods for study selection, data extraction, and analysis, and performing the subgroup analyses and sensitivity analyses.

In conclusion, the partial omentectomy had advantages in reducing operation time and trended to decrease intraoperative blood loss. And the survival in patients with partial omentectomy seemed to be comparable to that of patients with total omentectomy. Comparisons of the survival and safety between total omentectomy and partial omentectomy for gastric cancer needs to be further explored in large-scale RCTs.

## Data Availability Statement

The original contributions presented in the study are included in the article/supplementary material, further inquiries can be directed to the corresponding author.

## Author Contributions

Y-XZ and H-DL: study conception and design, acquisition of data, and drafting of manuscript. Y-XZ, Z-HC, and TJ: analysis and interpretation of data. KY and J-KH: critical revision of manuscript. All authors contributed to the article and approved the submitted version.

## Funding

Domestic support from (1) National Natural Science Foundation of China (No. 81772547); (2) the Fundamental Research Funds for the central Universities (No. 2017SCU04A18); (3) Young scientific and academic leaders training program of Sichuan University (No. 0082604151001/035); (4) Foundation of Science and Technology Department of Sichuan Province (No. 2019YFS0256); (5) 1. 3. 5 project for disciplines of excellence, West China Hospital, Sichuan University (No. ZY2017304).

## Conflict of Interest

The authors declare that the research was conducted in the absence of any commercial or financial relationships that could be construed as a potential conflict of interest.

## Publisher's Note

All claims expressed in this article are solely those of the authors and do not necessarily represent those of their affiliated organizations, or those of the publisher, the editors and the reviewers. Any product that may be evaluated in this article, or claim that may be made by its manufacturer, is not guaranteed or endorsed by the publisher.

## Abbreviations

PO: partial omentectomy
TO: total omentectomy
NOS: Newcastle-Ottawa Scale
RCT: randomized controlled trial
WMD: weighted mean difference
OR: odds ratio
OS: overall survival
DFS: disease-free survival
RFS: recurrence free survival
CI: confidence intervals.
